# Prevalence and Factors Related to Physical Activity in Spanish Adults with Obesity and Overweight: Analysis of the European Health Surveys for the Years 2014 and 2020

**DOI:** 10.3390/healthcare12141382

**Published:** 2024-07-10

**Authors:** Clara Maestre-Miquel, Ana López-de-Andrés, Napoleón Perez-Farinos, Ana Jimenez-Sierra, Juan Carlos Benavente-Marin, Ángel López-González, Antonio Viñuela-Sanchez, Rodrigo Jiménez-Garcia

**Affiliations:** 1 Departamento de Enfermería, Fisioterapia y Terapia Ocupacional, Universidad de Castilla-la Mancha, 13001 Ciudad Real, Spain; clara.maestre@uclm.es (C.M.-M.); angel.lopez@uclm.es (Á.L.-G.); antonio.vinuela@uclm.es (A.V.-S.); 2 Department of Public Health and Maternal & Child Health, Faculty of Medicine, Universidad Complutense de Madrid, 28040 Madrid, Spain; rodrijim@ucm.es; 3 EpiPHAAN Research Group, Universidad de Málaga–Instituto de Investigación Biomédica de Málaga (IBIMA), 29590 Málaga, Spain; napoleon.perez@uma.es (N.P.-F.); jc.benaventemarin@gmail.com (J.C.B.-M.); 4 Faculty of Medicine, Universidad San Pablo CEU, 28003 Madrid, Spain; a.jimenez100@usp.ceu.es

**Keywords:** physical activity, obesity, overweight, survey, Spain

## Abstract

(1) Background: To analyze the prevalence of physical activity (PA) according to the presence of overweight or obesity and other sociodemographic factors in the Spanish adult population. (2) Methods: Cross-sectional study using the European Health Interview Surveys for Spain from 2014 and 2020. (3) Results: In overweight and obese people, the percentage of those who reported not performing any type of PA remained constant between 2014 and 2020, while a statistically significant increase was observed in the percentage of people who walked for 10 min a day and exercised at least 2 days a week. The probability of being obese with respect to normal weight was higher in individuals who reported not engaging in PA during leisure time (OR 1.42; 95% CI 1.31–1.53), those who did not walk 10 min a day at least 2 days a week (OR 1.25; 95% CI 1.15–1.35), and those who did not exercise at least 2 days a week (OR 1.42; 95% CI 1.32–1.53). The probability of being overweight was higher in individuals who reported not performing PA during leisure time (OR 1.07; 95% CI 1.02–1.15) and in those who did not exercise at least 2 days per week (OR 1.15; 95% CI 1.09–1.22). (4) Conclusions: Small increases in PA have been observed in both overweight and obese individuals from 2014 to 2020.

## 1. Introduction

Overweight and obesity are multifactorial conditions involving biological, psychosocial, socioeconomic, and environmental components [1,2]. In turn, they are associated with noncommunicable diseases such as type II diabetes [3], hypertension, stroke, cardiovascular disease [1], and some types of cancer [4], all of which generate a very high economic cost for the health system [5].

Obesity is the most prevalent chronic disease worldwide [6] and is clearly increasing in frequency [7]. According to recent studies comparing various countries in Europe, approximately half of the European population is overweight or obese, and this is especially visible in women and persons with a low socioeconomic status [8].

Regular physical activity (PA) is associated with positive effects against excess weight [9] and even long-term obesity [10]. However, global PA levels have declined in recent decades [11]. In Spain, 36.41% of adults report spending their free time almost entirely sedentary [12].

Despite global recommendations on regular PA [13], there is still a need for improvement in public health interventions. In Spain, the PREDIMED-Plus trial, which evaluated the effect of an intervention with weight loss goals in 6872 participants, observed an increase in sedentary lifestyle, especially in people with higher body mass index (BMI), as well as an association between sedentary lifestyle and metabolic syndrome [14]. Therefore, in a population with a higher BMI, PA seems to play an important role in the prevention of associated comorbidities.

Recent reviews of the literature show that the programs that are most successful in the management of obesity are those in which the condition has been treated not only through regular PA but also through education on food and nutrition. Thus, significant improvements have been found in functional capacity, eating habits, weight loss, physical condition, and even in plasma variables [15,16].

Determining PA levels in overweight or obese adults is of special interest when designing future strategies for health promotion and disease prevention in this group. Therefore, the aim of this study was to analyze the association between PA and obesity/overweight in the Spanish adult population through the European Health Interview Surveys for Spain (EHISS) from 2014 and 2020. We specifically analyzed PA according to weight categories and the effect of gender, age, and other sociodemographic and clinical variables on adherence to PA. Finally, we analyzed the risk of overweight and obesity with respect to normal weight based on the various sociodemographic and health variables included in the study.

## 2. Materials and Methods

### 2.1. Study Design and Data Source

The study was cross-sectional with descriptive and analytical components and based on data from the 2014 and 2020 EHISS [17].

The EHISS is carried out approximately every 4 years and collects self-reported information on health in a representative sample of individuals aged 15 years and over residing in Spain. This information includes sociodemographic characteristics, self-reported illness, use of medications and health services, and lifestyle. Participants were selected in 3 stages. The first-stage units are the census tracts (37,000), which are grouped into 7 strata according to the size of the municipality in which they are located. Second-stage units are dwellings, which include all residents in the household. Third-stage units are individuals (1 for each household) who are randomly selected from all residents aged 15 years and over in the household. The surveys were carried out in the home by means of a computer-assisted personal interview and were supplemented, if necessary, by telephone interviews. Interviews were conducted over a 12-month period, from January to December 2014 for EHISS2014 and from July 2019 to July 2020 for EHISS2020. Efforts were made to ensure that interviews were conducted throughout the year in such a way that the number of participants was homogeneous every week and that all periods of the year were equally represented. It is important to note that from March to July 2020, interviews were conducted by telephone owing to the SARS-CoV-2 pandemic. Further details on the surveys can be found on the website of the National Institute of Statistics [18].

### 2.2. Population and Study Variables

The study was based on data from participants aged 18 to 104 years in EHISS2014 and in EHISS2020.

Three variables were used to evaluate PA: “Frequency of PA in leisure time”, “Number of days per week an individual goes walking for at least 10 min”, and “Number of days per week an individual does sport”. For the first variable, the survey asks how often participants engage in PA during their free time. Those who responded, “I don’t exercise. Leisure time was occupied in a sedentary manner” were classified as “No-PA”; and those who answered “Occasionally”, “I do PA several times a month”, or “I do sports or go training several times a week” were classified as “Occasional or frequent PA”. For the second and third variables, questions were used that asked how many days a week they walked 10 min at a time and did sport, respectively. Both variables were categorized into “One or no days” and “Two or more days”.

Weight was assessed using the body mass index (BMI), which was calculated based on self-reported weight and height. BMI was categorized into “normal weight” (BMI between 18.5 and <25 kg/m^2^), “overweight” (BMI between 25 and < 30 kg/m^2^), and “obesity” (BMI ≥ 30 kg/m^2^). Participants who did not answer the weight and/or height question or with BMI < 18.5 were excluded from the study.

In addition to the variables age, gender, and cohabitation as a couple (No/Yes), the maximum level of education was evaluated. To this end, a variable with 3 categories (primary or no education, secondary education, higher education) was developed based on the information from the survey.

Health information included self-perceived health and some self-reported chronic diseases (chronic obstructive pulmonary disease (COPD), diabetes, cardiovascular disease, stroke, cancer, mental illness, and high blood pressure). Three questions were used for each disease: 1. Have you ever had the disease? 2. Have you suffered from the disease in the last 12 months? 3. Was the disease diagnosed by a doctor? Participants who answered yes to all 3 questions were classified as “Yes”. In addition, data were recorded on health limitations (if any) and the extent of these limitations during the previous 6 months. Active smoking and alcohol consumption were also collected. The questions used to create all these variables are detailed in Table S1.

### 2.3. Statistical Analysis

Participants were described according to the study variables. Quantitative variables were expressed as mean and standard deviation (SD) and qualitative variables as frequencies and percentages. The assumption of normality of the quantitative variables was tested using the Kolmogorov–Smirnov test.

We evaluated whether there were differences in the study variables between the EHISS2014 and EHISS2020 depending on the weight category, using the chi-square test for the qualitative variables and the t test for independent samples in the quantitative variables.

We evaluated whether there was an association between weight category (normal weight, overweight, or obesity) and the 3 PA variables using the chi-square test. We stratified the analysis by the independent variables.

To assess whether PA was independently associated with weight category, odds ratios (ORs) with 95% confidence intervals (95% CIs) were calculated using a multivariable multinomial logistic regression model in which the dependent variable was weight categories and the category “normal weight” was a reference category. The independent variables were the 3 PA variables. In addition, other variables that were potentially associated with weight category were included in the models. To be included, the variables had to show a statistically significant bivariate association with weight category or be considered relevant after a review of the scientific literature. Wald’s test was used to decide which variables remained in the model. Various interactions between variables were tested.

The statistical analysis was performed with the program IBM SPSS Statistics for Windows, Version 27.0 (IBM Corp., Armonk, NY, USA).

### 2.4. Ethical Aspects

All participants in the EHISS2014 and EHISS2020 gave their informed consent to participate in the surveys.

The anonymized databases of the EHISS2014 and EHISS2020 are freely accessible and free to download from the Spanish Ministry of Health website. According to Spanish law, approval by an ethics committee is not necessary for epidemiological studies based on anonymized publicly accessible data [17].

## 3. Results

The data used in this study were from the 20,178 participants aged 18 years or over in the EHISS2014 and 19,826 participants in the EHISS2020. Table 1 shows the distribution of the study variables in overweight and obese individuals in the two surveys analyzed. Among overweight individuals, 61.8% perceived their health to be good or very good in 2014, and this percentage rose significantly to 65.8% in 2020. The percentage of people who reported having COPD, diabetes, and mental illness decreased significantly between 2014 and 2020, while the percentage of individuals with cancer increased. In 2014, 53.4% of people with obesity considered their health to be good or very good, and in 2020 this percentage had increased to 58.0% (*p* < 0.001). Between 2014 and 2020, only the percentages of people with mental illness decreased significantly, whereas the percentage of people with cancer also increased.

The prevalence of smoking and alcohol consumption decreased from 2014 to 2020 in participants with overweight and obesity. Also, in both groups, the percentage of those who reported no PA remained constant between 2014 and 2020, while the percentage of people who walked 10 min a day and exercised at least 2 days a week increased significantly.

### 3.1. Differences by Gender and Age in Self-Reported PA in People with Obesity

**Figure 1:**
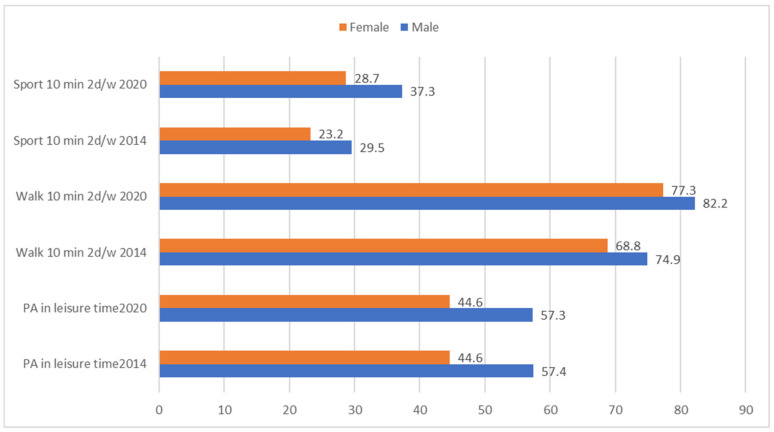
Proportion of men and women with obesity who reported doing PA in free time or reported doing sports or walking 10 min/2 or more days per week, in 2014 and 2020.

Figure 1 shows that, among the obese population, a higher percentage of men than women (all *p* < 0.001) reported some PA in their leisure time (57.4% vs. 44.6% in 2014 and 57.3% vs. 44.6% in 2020), walking 10 min a day at least 2 days a week (74.9% vs. 68.8% in 2014 and 82.2% vs. 77.3% in 2020), and doing sport at least 2 days a week (29.5% vs. 23.2% in 2014 and 37.3% vs. 28.7% in 2020).

**Figure 2:**
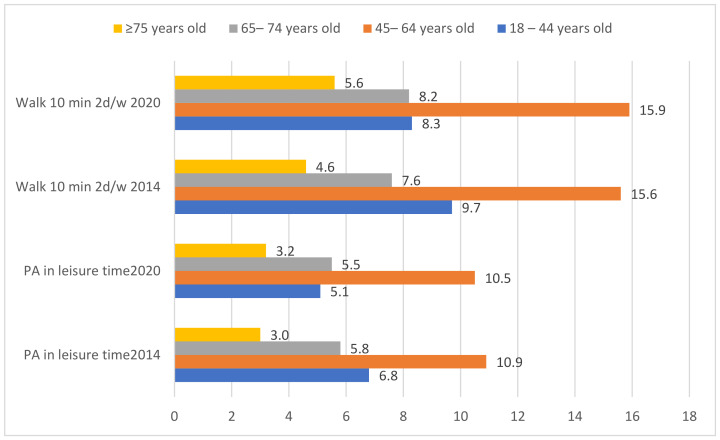
Differences by age group between obese people reported doing PA in free time or reported doing sports or walking 10 min/2 or more days per week, in 2014 and 2020.

Figure 2 shows that among obese people, the age group in which PA is most frequent during leisure time and participants walk 10 min a day and do sport at least 2 days a week is those aged 45 to 64 years, both in 2014 and 2020. The difference for the other age groups was significant in all cases.

### 3.2. Differences in Reported PA between Normal-Weight, Overweight, and Obese Participants

Table 2 shows the prevalence of PA variables stratified by sociodemographic characteristics and weight categories. The percentage of people who reported PA (walked) and did sports at least 2 days a week was significantly higher (*p* < 0.05) in people with normal weight and lower in people with obesity in all categories of the variables analyzed. Among men with normal weight, 72.9% reported some PA during their leisure time, 84.5% walked 10 min a day at least 2 days a week, and 52.8% exercised at least 2 days a week. Among men who were obese, these percentages were significantly lower (*p* < 0.001), namely, 57.4%, 78.4%, and 33.3%, respectively. A similar trend was found in women. Among women with normal weight, 65.3% reported some PA during their leisure time, 84.3% walked 10 min a day at least 2 days a week, and 45.1% did sports at least 2 days a week; among obese women, the percentages were significantly lower (*p* < 0.001), namely, 44.6%, 72.8%, and 25.8%, respectively.

The percentage of people who reported PA in their leisure time, walking, and exercising at least 10 min a week was significantly higher (*p* < 0.05) in men, in younger individuals, and in those with a higher educational level in all three weight categories.

The percentage of people reporting PA and who walked and exercised at least 2 days a week was significantly higher (*p* < 0.05) in people with normal weight and lower in people with obesity in all categories of all clinical and lifestyle variables (Table 3). Likewise, people who perceived themselves as being in “good or very good” health or who did not have any of the clinical conditions analyzed reported performing PA in their leisure time, walking, and doing sport at least 10 min a week in all three weight categories.

### 3.3. Results of the Multinomial Logistic Regression Analysis to Identify PA Variables and Variables Independently Associated with Overweight and Obesity

As can be seen in Table 4, after adjusting for the multivariate model, there is an association between weight class and PA. Thus, the probability of being obese with respect to normal weight was higher in individuals who reported no PA during their leisure time (OR 1.42; 95% CI 1.31–1.53), those who did not walk 10 min a day at least 2 days a week (OR 1.25; 95% CI 1.15–1.35), and those who did not exercise at least 2 days a week (OR 1.42; 95% CI 1.32–1.53). In addition, the probability of being overweight with respect to normal weight was higher in individuals who reported no PA during their leisure time (OR 1.07; 95% CI 1.02–1.15) and in those who did not exercise at least 2 days per week (OR 1.15; 95% CI 1.09–1.22).

The risk of overweight (OR 2.32; 95% CI 2.21–2.43) and obesity (OR 1.84; 95% CI 1.77–1.93) with respect to normal weight was higher in men than in women.

A higher risk of overweight and obesity compared to normal weight was observed in people with a lower level of education, in people who lived with a partner, and in people with asthma, diabetes mellitus, and high blood pressure. Smokers showed a lower risk of overweight and obesity than persons with normal weight.

## 4. Discussion

When we compared two independent and representative samples of the Spanish adult population obtained in the years 2014 and 2020, we found that, among overweight and obese people, the prevalence of PA remained constant between 2014 and 2020, while the percentage of people who walked 10 min a day and exercised at least 2 days a week increased significantly. These results are consistent with trends in leisure-time PA among the Spanish general population, which improved slightly from 1987 to 2020 [19]. A contrary trend has been reported in other countries, where the proportion of people with obesity and sedentary behaviors increased significantly during the same period [20]. This could be due to differences in the measurement of PA according to national health surveys, as well as sociocultural and environmental factors.

Previous research has shown differences in indicators of leisure-time PA and its relationship with BMI between men and women [21,22,23,24,25,26,27,28]. According to the 2022 Living Conditions Survey in Spain [23], the percentage of men who do regular physical exercise in their free time is higher than that of women. Also in Spain, 11,883 NUTRiMDEA cohort participants were interviewed using the International Physical Activity Questionnaire, finding a significantly higher metabolic equivalent value minutes per week among men than women (2910 vs. 2207; *p* < 0.001) [26]. In the US, the analysis of the National Health and Nutrition Examination Survey (NHANES) between 2009 and 2018 reported that males had a higher PA score (70.6% vs. 54.9%, *p* < 0.001) than females [27]. Guthold R. et al. conducted a pooled data study of population-based surveys investigating the prevalence of insufficient PA, which included PA at work, at home, for transport, and during leisure time (i.e., not doing at least 150 min of moderate-intensity, or 75 min of vigorous-intensity PA per week, or any equivalent combination of the two). A total of 358 surveys, across 168 countries, including 1.9 million participants, were analyzed finding that the global age-standardized prevalence of insufficient PA was 31.7% (95% CI 28.6–39.0) in women and 23.4%, (95% CI 21.1–30.7) in men [28].

Our results are similar: they indicate a higher percentage of men than women with obesity who reported PA in their free time. According to some, the reasons for physical exercise tend to differ by gender: while men are motivated by competitive activities, women tend to be motivated by aesthetic aspects [29], possibly explaining why the association with gender differences goes beyond personal attitudes and motivations, affecting areas such as lifestyle and other social determinants of health. In this sense, the association between female gender and low socioeconomic status, sedentary lifestyle, and obesity has been shown in other countries [30,31,32,33], as has the increase in inequalities in obesity according to educational level in women but not in men [34]. This would explain, in part, the more frequent sedentary leisure we observed among obese women than among obese men.

In general, PA levels are lower in overweight and obese populations than in people with normal weight, as demonstrated by studies in Spain [35] and elsewhere [33,36]. Our results are consistent with the findings of these studies.

We found that 33.3% of men and 25.8% of women reported doing sport at least 2 days per week. While measurement of PA in leisure time differs according to the study, the percentages are clearly similar. Zhang et al. [37] report figures of up to 30.6% for obese individuals who declared themselves to be physically active. Nevertheless, this finding remains controversial. While some authors report very poor levels of PA in the obese population (less than 16 min a day, of which less than 3 min was vigorous activity) [38] or even no activity in a high percentage of this group (78.2% in men and 68.3% in women) [39], we identified a study that shows high levels of PA among obese people (up to 47% of obese individuals performed more than 300 min per week of moderate–vigorous activity) [13]. In terms of its effects, metabolically healthy obesity has been described in the most active individuals, in contrast with metabolically unhealthy obesity in the most sedentary [40], with “metabolically healthy obesity” defined as normal blood pressure, adequate cholesterol levels, relatively low visceral adipose mass, and preserved insulin sensitivity. “Metabolically unhealthy obesity” is the term used for persons with multiple cardiovascular risk factors [41], although the definition has been scientifically disputed [42].

There is a circular relation between having obesity and not moving or not moving and acquiring overweight and obesity. Furthermore, being physically active can contribute to a metabolically healthy profile even in the presence of obesity. However, metabolically healthy obesity is a transient condition, and PA alone may not be sufficient for its maintenance [43].

The effect of age and other sociodemographic and clinical variables on adherence to PA were also considered in this study. The profile we found for active people, regardless of BMI, was younger males with a high educational level. In this regard, PA is expected to decrease with age owing to biological mechanisms [44]. In terms of educational level, our results are in line with others highlighting increases in PA with educational level in obese people [45].

Studies conducted in Spain, Germany, the US, and Australia, among others countries, have reported that greater adherence to PA practice (action and maintenance stages) was related to better academic level and higher economic income [46,47,48,49]. This was confirmed in a literature review conducted by O’Donoghue et al., reporting that low socioeconomic status (low educational levels and low economic income) was comprised of factors associated to low levels of PA [50].

Our results show that a high proportion of individuals with overweight (65.8%) and obesity (58.0%) self-perceived their health as good or very good. Previous studies conducted in our country show that most participants in health surveys tend to respond that their health is good or even very good. In fact, data of the EHISS2020 showed that over 75% of the general population considered their health as good or very good [18]. According to recent data of the World Health Organization, the mean percentage of the population living in member countries of the European Union who self-assessed their health as good or very good was 66.5%. The equivalent figure for Spain in that database was almost 7% higher (72.4%) [51].

The possible overrating of self-perceived health in population surveys, beside obesity status, can be in part caused by cultural factors and social desirability of answers [52]. Furthermore, previous investigations conducted in Spain agree in finding high prevalence of good self-rated health among people with overweight and obesity [53,54].

We observed an increased risk of overweight and obesity in people who live with a partner. Tzotzas et al. reported that among Greek adults, marital status was significantly associated with obesity and abdominal obesity status in both genders [55]. Furthermore, marriage or living in a couple contributes to an increased risk of overweight and obesity in other countries [56,57,58]. Future investigations must confirm this relationship and explore possible explanations.

Given these results, it seems necessary to redouble efforts to promote regular PA in the obese and overweight population. A recent review of the literature reports that interventions in overweight or obese adults currently involve PA, diet, or both, with the common goal of weight loss and improved quality of life [59].

The Scientific Committee of the Spanish Agency for Food Safety and Nutrition [60] states that any amount of PA is better than none and concurs with the WHO recommendations of at least 150 to 300 min of moderate aerobic activity per week (or the equivalent, i.e., between 75 and 150 min of vigorous activity) for all adults. Given that an association has been found between increase in resistance exercise and reduction in body mass, specifically in the obese population, and not as much with aerobic exercise [61], it would be interesting to investigate resistance exercises in this population, without neglecting other types of PA. In fact, current recommendations for lowering and maintaining body weight include muscular resistance training as part of the exercise prescribed. In the case of excess weight and obesity, the American College of Sport Medicine [62] recommends a minimum of 200 to 300 min of moderate- to vigorous-intensity PA per week. Gutt et al. [63] stress the importance of progressive prescribing in adults with obesity, starting with at least 150 min per week of moderate-intensity aerobic PA and progressively adding combined muscular endurance exercises that involve large muscle groups. It is necessary to insist on the long-term maintenance of PA habits to ensure that they yield optimal results.

In our opinion, beside the recommendations made by Scientific Societies and public health authorities, several special considerations are important for improving PA among people with overweight and obesity. First, prevalent obesity-related comorbidities must be considered before prescribing exercise to maximize patient safety. Second, the recommendation for these populations is to “start low and go slow”. Third, it is a good idea to spread out aerobic activity over the week versus all the time in one day. Fourth, it is recommended to use wearable exercise trackers such as smartwatches, cellular smartphones, pedometers, heart rate monitors, etc. Physicians and patients could potentially use these technological advances to improve their relationships further. Also, utilizing technology to have doctor–patient check-ins regarding their exercise may increase the adherence of obese individuals to exercise programs. Fifth, nurses, physicians, and anyone else involved in the healthcare setting with obese patients should employ motivational interviewing techniques to ensure that patients are meeting their exercise goals [64,65].

Our study is limited by its cross-sectional design, which prevents us from establishing causality. Moreover, since self-reporting of weight and height has not been validated in the EHISS, the proportion of obese people may have been underestimated in our study [66]. Even so, surveys with self-reported BMI data have been used very frequently in epidemiological research [67,68] owing to the strong correlation between self-reported data and measured data [69].

The usefulness of health surveys in collecting information on PA has been addressed in various studies [70,71,72]. However, they are affected by the need to use more than a single question and the possibility of recall and social desirability biases and selection bias due to nonparticipation. These biases have been shown to result in overestimates of PA and underestimates of sedentary behavior [70,71,72].

As strengths, it is worth highlighting the use of lifestyle variables and self-perception of health and multiple sociodemographic variables that cannot be obtained from medical records. In addition, the EHISS is a structured survey that enables the comparison of two periods with a large sample.

Only small increases in PA were observed among overweight and obese individuals between 2014 and 2020. The prevalence of PA measured with the three questions was lower in overweight and obese individuals than in those with normal weight. Women engage in PA less frequently than men in all weight classes.

## 5. Conclusions

In overweight and obese people, the percentage of those who reported not performing any type of PA remained constant between 2014 and 2020. PA levels were lower in overweight and obese populations than in people with normal weight. Being a man, good self-perceived health, younger age, and a high educational level were variables associated to more PA among obese and overweight people.

Given these results, we consider that the planning and implementation of moderate-to-intense PA strategies on a regular basis is of vital importance for overweight and obese populations, as well as for the prevention of obesity, specifically in patients with previous conditions associated with an increased risk of this disease. Finally, health equity strategies should be encouraged, as they cushion the impact of socioeconomic factors on people with higher BMIs.

## Figures and Tables

**Table 1:** Distribution according to study variables of people with overweight or obesity included in the European Health Interview Surveys for Spain (EHISSs) conducted in 2014 and 2020.

		Overweight (BMI ≥ 25 kg/m^2^)					Obesity (BMI ≥ 30 kg/m^2^)				
		2014		2020			2014		2020		
		n	%	n	%	*p*	n	%	n	%	*p*
Gender	Male	6272	54.5	6240	55.2	0.308	1778	48.6	1687	50.4	0.116
	Female	5234	45.5	5068	44.8		1884	51.4	1658	49.6	
Age	Mean (SD)	56.5	16.8	58.2	16.6	<0.001	57.9	16.3	58.9	16.0	0.007
	18–44 years old	3168	27.5	2593	22.9	<0.001	879	24	688	20.6	0.008
	45–64 years old	4368	38	4462	39.5		1419	38.7	1352	40.4	
	65–74 years old	2049	17.8	2158	19.1		723	19.7	692	20.7	
	≥75 years old	1921	16.7	2095	18.5		641	17.5	613	18.3	
Level of education	Primary/no education	7198	62.6	6607	58.4	<0.001	2581	70.5	2160	64.6	<0.001
	Secondary education	1954	17	2098	18.6		518	14.1	581	17.4	
	Higher education	2354	20.5	2603	23.0		563	15.4	604	18.1	
Living with a partner	No	4604	40	4953	43.8	<0.001	1519	41.5	1455	43.5	0.088
	Yes	6902	60	6355	56.2		2143	58.5	1890	56.5	
Physical activity in leisure time	No	4610	40.1	4568	40.4	0.611	1801	49.2	1639	49.0	0.879
	Yes	6896	59.9	6740	59.6		1861	50.8	1706	51.0	
Walking for 10 min at least 2 days a week	No	2649	23.0	1816	16.1	<0.001	1033	28.2	678	20.3	<0.001
	Yes	8857	77.0	9492	83.9		2629	71.8	2667	79.7	
Sport at least 2 days a week	No	7493	65.1	6810	60.2	<0.001	2701	73.8	2239	66.9	<0.001
	Yes	4013	34.9	4498	39.8		961	26.2	1106	33.1	
Self-perceived health	Fair/poor/very poor	4399	38.2	3868	34.2	<0.001	1707	46.6	1406	42.0	<0.001
	Very good/good	7107	61.8	7440	65.8		1955	53.4	1939	58.0	
Limitations during the previous 6 months	No	7145	77.9	6706	78.8	0.007	2172	59.3	2070	61.9	0.038
	Mild	1600	17.4	1464	17.2		1142	31.2	1005	30.0	
	Severe	425	4.6	342	4.0		347	9.5	270	8.1	
Chronic obstructive pulmonary disease	No	10,848	94.3	10,772	95.3	0.001	3384	92.4	3118	93.2	0.193
	Yes	658	5.7	536	4.7		278	7.6	227	6.8	
Diabetes mellitus	No	10,848	94.3	10,772	95.3	0.028	3050	83.3	2758	82.5	0.353
	Yes	658	5.7	536	4.7		612	16.7	587	17.5	
Cardiovascular disease	No	10,134	88.1	10,040	88.8	0.093	3112	85	2856	85.4	0.638
	Yes	1372	11.9	1268	11.2		550	15	489	14.6	
Stroke	No	11,217	97.5	11,052	97.7	0.220	3568	97.4	3259	97.4	0.991
	Yes	289	2.5	256	2.3		94	2.6	86	2.6	
Cancer	No	10,986	95.5	10,692	94.6	0.001	3496	95.5	3134	93.7	0.001
	Yes	520	4.5	616	5.4		166	4.5	211	6.3	
High blood pressure	No	7454	64.8	7232	64.0	0.191	2040	55.7	1845	55.2	0.643
	Yes	4052	35.2	4076	36.0		1622	44.3	1500	44.8	
Mental illness	No	9614	83.6	9711	85.9	<0.001	2931	80	2745	82.1	0.031
	Yes	1892	16.4	1597	14.1		731	20	600	17.9	
Smoking	No	8975	78.0	9058	80.1	<0.001	2897	79.1	2746	82.1	0.002
	Yes	2531	22.0	2250	19.9		765	20.9	599	17.9	
Alcohol	No	4959	43.1	5801	51.3	<0.001	1556	42.5	1736	51.9	<0.001
	Yes	6547	56.9	5507	48.7		2106	57.5	1609	48.1	

**Table 2:** Prevalence of physical activity in leisure time, engagement to walking ≥ 2 days per week and to practice sport ≥2 days per week among subjects with normal weight, overweight, and obesity and gender–age-matched subjects according to sociodemographic variable.

	Physical Activity in Leisure Time							Walking for 10 min at Least 2 Days a Week							Sport at Least 2 Days a Week						
	Normal Weight		Overweight		Obesity			Normal Weight		Overweight		Obesity			Normal Weight		Overweight		Obesity		
	n	%	n	%	n	%	*p* ^a^	n	%	n	%	n	%	*p* ^b^	n	%	n	%	n	%	*p* ^c^
Gender ^d,e,f,g,h,i,j,k,l^																					
Male	5094	72.9	6197	68.5	1988	57.4	<0.001	5909	84.5	7551	83.5	2718	78.4	<0.001	3689	52.8	3984	44.0	1154	33.3	<0.001
Female	6993	65.3	3872	57.3	1579	44.6	<0.001	9023	84.3	5502	81.4	2578	72.8	<0.001	4829	45.1	2460	36.4	913	25.8	<0.001
Age groups ^d,e,f,g,h,i,j,k,l^																					
18–44 years old	5685	71.4	2836	67.6	836	53.4	<0.001	6812	85.5	3510	83.7	1261	80.5	<0.001	4349	54.6	2080	49.6	548	35.0	<0.001
45–64 years old	4074	70.0	4060	67.0	1502	54.2	<0.001	4984	85.6	5148	85.0	2208	79.7	<0.001	2832	48.6	2609	43.1	889	32.1	<0.001
65–74 years old	1346	72.3	1929	69.1	793	56.0	<0.001	1648	88.6	2429	87.0	1111	78.5	<0.001	856	46.0	1151	41.2	421	29.8	<0.001
≥75 years old	982	48.1	1244	45.0	436	34.8	<0.001	1488	72.9	1966	71.2	716	57.1	<0.001	481	23.6	604	21.9	209	16.7	<0.001
Level of education ^d,e,f,g,h,i,j,k,l^																					
Primary/no education	4328	58.7	5153	56.9	2212	46.7	<0.001	5987	81.2	7259	80.1	3445	72.7	<0.001	2609	35.4	2936	32.4	1183	25.0	<0.001
Secondary education	2745	70.9	2067	70.0	634	57.7	<0.001	3381	87.3	2543	86.1	895	81.4	<0.001	2063	53.3	1438	48.7	396	36.0	<0.001
Higher education	5014	77.8	2849	75.2	721	61.8	<0.001	5564	86.4	3251	85.8	956	81.9	<0.001	3846	59.7	2070	54.6	488	41.8	<0.001
Living with a partner ^e,f,g,i,j,k^																					
No	5896	68.4	4086	62.1	1438	48.4	<0.001	7359	85.3	5431	82.5	2181	73.3	<0.001	4304	49.9	2592	39.4	843	28.3	<0.001
Yes	6191	68.3	5983	64.9	2129	52.8	<0.001	7573	83.5	7622	82.6	3115	77.2	<0.001	4214	46.5	3852	41.8	1224	30.3	<0.001

^a^ *p*-value for the difference in the percentages of individuals who perform some PA between individuals with normal weight, overweight, and obesity (Chi-squared). ^b^ *p*-value for the difference in the percentages of individuals who walk 10 min a day at least 2 days a week between individuals with normal weight, overweight, and obesity (Chi-squared). ^c^ *p*-value for the difference in the percentages of individuals who do sports at least 2 days a week between individuals with normal weight, overweight, and obesity (Chi-squared). ^d^ Significant difference in the percentage of individuals who perform some PA between the categories of the variable, in those who have normal weight (Chi-squared). ^e^ Significant difference in the percentage of individuals who walk 10 min at least 2 times a week between the categories of the variable, in those who have normal weight (Chi-squared). ^f^ Significant difference in the percentage of individuals who do sports at least 2 times a week between the categories of the variable, in those who have normal weight (Chi-squared). ^g^ Significant difference in the percentage of individuals who perform some PA between the categories of the variable, in those who are overweight (Chi-squared). ^h^ Significant difference in the percentage of individuals who walk 10 min at least 2 times a week between the categories of the variable, in those who are overweight (Chi-squared). ^i^ Significant difference in the percentage of individuals who do sports at least 2 times a week between the categories of the variable, in those who are overweight (Chi-squared). ^j^ Significant difference in the percentage of individuals who perform some PA between the categories of the variable, in those who have obesity (Chi-squared). ^k^ Significant difference in the percentage of individuals who walk 10 min at least 2 times a week between the categories of the variable, in those who are obese (Chi-squared). ^l^ Significant difference in the percentage of individuals who do sports at least 2 times a week between the categories of the variable, in those who have obesity (Chi-squared).

**Table 3:** Prevalence of physical activity in leisure time, engagement to walking ≥ 2 days per week and to practice sport ≥2 days per week among subjects with normal weight, overweight, and obesity and gender–age-matched subjects according to clinical variables and lifestyles.

	Physical activity in Leisure Time							Walking for 10 min at Least 2 Days a Week							Sport at Least 2 Days a Week						
	Normal Weight		Overweight		Obesity			Normal Weight		Overweight		Obesity			Normal Weight		Overweight		Obesity		
	n	%	n	%	n	%	*p* ^a^	n	%	n	%	n	%	*p* ^b^	n	%	n	%	n	%	*p* ^c^
Self-perceived health ^d,e,f,g,h,i,j,k,l^																					
Fair/poor/very poor	2196	52.5	2584	50.1	1241	39.9	<0.001	3120	74.7	3830	74.3	2088	67.1	<0.001	1320	31.6	1409	27.3	684	22.0	<0.001
Very good/good	9891	73.2	7485	70.3	2326	59.7	<0.001	11812	87.4	9223	86.6	3208	82.4	<0.001	7198	53.3	5035	47.3	1383	35.5	<0.001
Limitations during the previous 6 months ^d,e,f,g,h,i,j,k,l^																					
No	10088	72.8	7753	69.2	2498	58.9	<0.001	12114	87.5	9729	86.8	3500	82.5	<0.001	7287	52.6	5171	46.1	1479	34.9	<0.001
Mild	1760	57.4	2055	55.7	932	43.4	<0.001	2442	79.7	2898	78.5	1530	71.3	<0.001	1102	36.0	1121	30.4	509	23.7	<0.001
Severe	231	30.1	258	28.6	136	22.0	0.002	231	30.1	258	28.6	136	22.0	0.157	231	30.1	258	28.6	136	22.0	0.112
Diabetes mellitus ^d,e,f,g,h,i,j,k,l^																					
No	11596	69.1	9192	64.8	3040	52.3	<0.001	14234	84.8	11816	83.3	4482	77.2	<0.001	8250	49.2	5962	42.0	1782	30.7	<0.001
Yes	491	54.0	877	53.9	527	44.0	<0.001	698	76.7	1237	76.1	814	67.9	<0.001	268	29.5	482	29.6	285	23.8	0.001
Chronic obstructive pulmonary disease ^d,e,f,g,h,i,j,k,l^																					
No	11800	69.0	9726	64.3	3379	52.0	<0.001	14511	84.8	12552	83.0	4991	76.8	<0.001	8349	48.8	6257	41.4	1969	30.3	<0.001
Yes	287	49.0	343	49.8	188	37.2	<0.001	421	71.8	501	72.7	305	60.4	<0.001	169	28.8	187	27.1	98	19.4	0.001
Cardiovascular disease ^d,e,f,g,h,i,j,k,l^																					
No	11486	69.4	9202	64.8	3142	52.6	<0.001	14100	85.1	11849	83.4	4656	78.0	<0.001	8184	49.4	5984	42.1	1845	30.9	<0.001
Yes	601	53.2	867	54.2	425	40.9	<0.001	832	73.7	1204	75.2	640	61.6	<0.001	334	29.6	460	28.7	222	21.4	<0.001
Stroke ^e,f,g,h,i,j,k,l^																					
No	11974	68.7	9889	64.0	3504	51.3	<0.001	14773	84.7	12817	83.0	5201	76.2	<0.001	8454	48.5	6360	41.2	2030	29.7	<0.001
Yes	113	44.1	180	49.3	63	35.0	0.007	159	62.1	236	64.7	95	52.8	0.027	64	25.0	84	23.0	37	20.6	0.555
Cancer ^d,e,f,g,h,i,j,k,l^																					
No	11686	68.6	9616	63.9	3402	51.3	<0.001	14395	84.6	12463	82.8	5046	76.1	<0.001	8284	48.7	6177	41.0	1975	29.8	<0.001
Yes	401	60.1	453	59.7	165	43.8	<0.001	537	80.5	590	77.7	250	66.3	<0.001	234	35.1	267	35.2	92	24.4	<0.001
High blood pressure ^d,e,f,g,h,i,j,k,l^																					
No	10452	69.8	7130	66	2054	52.9	<0.001	12718	85.0	9074	84.0	3076	79.2	<0.001	7546	50.4	4736	43.8	1252	32.2	<0.001
Yes	1635	60	2939	58.7	1513	48.5	<0.001	2214	81.3	3979	79.5	2220	71.1	<0.001	972	35.7	1708	34.1	815	26.1	<0.001
Mental illness ^d,e,f,g,h,i,j,k,l^																					
No	10968	70.1	8985	65.8	3004	52.9	<0.001	13406	85.7	11476	84.1	4423	77.9	<0.001	7835	50.1	5814	42.6	1760	31.0	<0.001
Yes	1119	54.9	1084	50.2	563	42.3	<0.001	1526	74.9	1577	73.1	873	65.6	<0.001	683	33.5	630	29.2	307	23.1	<0.001
Smoking ^d,f,g,i,j,l^																					
No	9161	70.7	8076	64.6	2924	51.8	<0.001	10969	84.6	10302	82.5	4263	75.6	<0.001	10969	84.6	10302	82.5	4263	75.6	<0.001
Yes	2926	61.9	1993	60.1	643	47.1	<0.001	3963	83.8	2751	83.0	1033	75.7	<0.001	1947	41.2	1229	37.1	361	26.4	<0.001
Alcohol ^d,e,f,g,h,i,j,k,l^																					
No	4560	60.0	3739	54.9	1593	44.4	<0.001	6227	81.9	5434	79.8	2594	72.3	<0.001	3031	39.9	2199	32.3	916	25.5	<0.001
Yes	7527	74.6	6330	70.4	1974	57.7	<0.001	8705	86.3	7619	84.7	2702	79.0	<0.001	5487	54.4	4245	47.2	1151	33.7	<0.001

^a^ *p*-value for the difference in the percentages of individuals who perform some PA between individuals with normal weight, overweight, and obesity (Chi-squared). ^b^ *p*-value for the difference in the percentages of individuals who walk 10 min a day at least 2 days a week between individuals with normal weight, overweight, and obesity (Chi-squared). ^c^ *p*-value for the difference in the percentages of individuals who do sports at least 2 days a week between individuals with normal weight, overweight, and obesity (Chi-squared). ^d^ Significant difference in the percentage of individuals who perform some PA between the categories of the variable, in those who have normal weight (Chi-squared). ^e^ Significant difference in the percentage of individuals who walk 10 min at least 2 times a week between the categories of the variable, in those who have normal weight (Chi-squared). ^f^ Significant difference in the percentage of individuals who do sports at least 2 times a week between the categories of the variable, in those who have normal weight (Chi-squared). ^g^ Significant difference in the percentage of individuals who perform some PA between the categories of the variable, in those who are overweight (Chi-squared). ^h^ Significant difference in the percentage of individuals who walk 10 min at least 2 times a week between the categories of the variable, in those who are overweight (Chi-squared). ^i^ Significant difference in the percentage of individuals who do sports at least 2 times a week between the categories of the variable, in those who are overweight (Chi-squared). ^j^ Significant difference in the percentage of individuals who perform some PA between the categories of the variable, in those who have obesity (Chi-squared). ^k^ Significant difference in the percentage of individuals who walk 10 min at least 2 times a week between the categories of the variable, in those who are obese (Chi-squared). ^l^ Significant difference in the percentage of individuals who do sports at least 2 times a week between the categories of the variable, in those who have obesity (Chi-squared).

**Table 4:** Risk of overweight and obesity according to a multinomial model adjusted for sociodemographic and health variables.

		Overweight			Obesity		
		OR	CI95%		OR	CI 95%	
European Health Interview Surveys	2014	1			1		
	2020	1.08	1.03	1.13	1.02	0.96	1.08
Gender	Male	1			1		
	Female	2.32	2.21	2.43	1.84	1.73	1.97
Age	18–44 years old	1			1		
	45–64 years old	1.65	1.56	1.75	1.58	1.46	1.71
	65–74 years old	1.80	1.66	1.95	1.40	1.26	1.55
	≥75 years old	1.39	1.28	1.52	0.70	0.62	0.78
Physical activity in leisure time	Yes	1			1		
	No	1.07	1.02	1.15	1.42	1.31	1.53
Walking for 10 min at least 2 days a week	Yes	1			1		
	No	1.01	0.95	1.07	1.25	1.15	1.35
Sport at least 2 days a week	Yes	1			1		
	No	1.15	1.09	1.22	1.42	1.32	1.53
Level of education	Primary/no education	1.63	1.54	1.72	2.33	2.15	2.53
	Secondary education	1.27	1.19	1.36	1.48	1.34	1.62
	Higher education	1			1		
Self-perceived health	Fair/poor/very poor	1.09	1.02	1.15	1.28	1.19	1.37
	Very good/good	1			1		
Living with a partner	No	1			1		
	Yes	1.24	1.18	1.30	1.23	1.16	1.30
Diabetes mellitus	No	1			1		
	Yes	1.22	1.11	1.34	1.76	1.59	1.94
Asthma	No	1			1		
	Yes	1.24	1.13	1.37	1.51	1.34	1.71
Cardiovascular disease	No	1			1		
	Yes	0.91	0.84	0.99	1.13	1.02	1.26
High blood pressure	No	1			1		
	Yes	1.87	1.76	1.99	2.94	2.73	3.17
Smoking	No	1			1		
	Yes	0.72	0.68	0.77	0.65	0.60	0.70

## Data Availability

The anonymized EHISS datasets are freely accessible and can be downloaded by anyone on the Ministry of Health’s website. https://www.sanidad.gob.es/estadEstudios/estadisticas/EncuestaEuropea/home.htm (accessed on 8 October 2023). All other relevant data are included in the paper.

## Supplementary Materials

The following supporting information can be downloaded at: https://www.mdpi.com/article/10.3390/healthcare12141382/s1, Table S1: Definition of variables according to the questions included in the European Health Interview Surveys in Spain conducted in the years 2014 and 2020.
