# Supplementary material for: Prevalence and Factors Related to Physical Activity in Spanish Adults with Obesity and Overweight: Analysis of the European Health Surveys for the Years 2014 and 2020
Source: Healthcare (Basel). 2024 Jul 10;12(14):1382. doi: 10.3390/healthcare12141382 (PMC11276508; doi:10.3390/healthcare12141382)
Supplement: Supplementary file 1 [file healthcare-12-01382-s001.zip › healthcare-3084517-supplementary.pdf]

Table S1. Definition of variables according to the questions included in the European Health Interview Surveys in Spain conducted in years 2014 and 2020.

**Questions**	**Description and answer**	**Variables name**	**Categories**
Which is your gender?	1. Male<br>2. Female	Gender	1. Men<br>2. Women
How old are you?	Age in years	Age groups	1. 15-54<br>2. 55-64<br>3. 65-74<br>4. ≥75
Has your doctor told you that you are suffering from diabetes?	1.Yes<br>2.No	Diabetes	1. Case<br>2. Control
Which of these possibilities best describes how often you do some physical activity in your free time?	1. I don't exercise. I occupy my free time almost completely sedentary.<br>2. I do some occasional physical or sports activity<br>3. I do physical activity several times a month<br>4. I do sports or physical training several times a week	Frequency of physical activity	1 . Sedentary or low Option 1 and 2<br>2. Medium or high physical activity Option 3 and 4
How many days, in a typical week, you do sports, gymnastics, bicycling, walking fast for at least 10 minutes continuously?	Number of days (0 to 7)	Number of days of physical activity per week	1. None or one day<br>2. Two or more days
What level of education have you completed?	1. Does not know how to read or write<br>2. Incomplete primary education<br>3. Complete primary education<br>4. First stage of Secondary Education, with or without a qualification<br>5. Elementary Spanish Upper Secondary Education<br>6. Upper secondary education<br>7. Intermediate vocational training or equivalent<br>8. Advanced vocational training or equivalent<br>9. University studies or equivalent<br>10. Over university (master, PhD….)	Educational level	1. No studies/Primary: Options 1 to 3<br>2. Secondary: Options 4 to 8<br>3. High education: Options 9 and 10
What is your marital status?	1. Single<br>2. Married<br>3. Widower<br>4. Separated<br>5. Divorced	Living with a partner	1. Yes: Option 2<br>2. Nor: options 1, 3, 4 and 5

Table S1. Definition of variables according to the questions included in the European Health Interview Surveys in Spain conducted in years 2014 and 2020. (Continued)

Questions	Description and answer	Variables	Categories
In the past twelve month, how is your perception of your general health status?	1. Very good<br>2. Good<br>3. Fair<br>4. Bad<br>5. Very bad	Self-rated health	1. Very good/good: Options 1 and 2<br>2. Fair/poor/very poor: Options 3 to 5
Has your doctor told you that you are suffering from COPD?	1.Yes<br>2.No	COPD	1. Yes<br>2. No
Has your doctor told you that you are suffering from heart diseases (heart failure or coronary disease?	1.Yes<br>2.No	Heart diseases	1. Yes<br>2. No
Has your doctor told you that you are suffering from stroke?	1.Yes<br>2.No	Stroke	1. Yes<br>2. No
Has your doctor told you that you are suffering from cancers?	1.Yes<br>2.No	Cancer	1. Yes<br>2. No
Has your doctor told you that you are suffering from anxiety or depression?	1.Yes<br>2.No	Mental disease	1. Yes<br>2. No
Has your doctor told you that you are suffering from High blood pressure?	1.Yes<br>2.No	High blood pressure	1. Yes<br>2. No
During the past 12 months, how often have you had alcoholic beverages of any kind (i.e. beer, wine, spirits, distilled and mixed drinks, or other alcoholic beverages)?	1. Daily or almost daily<br>2. 5-6 days per week<br>3. 3-4 days per week<br>4. 1-2 days per week<br>5. 2-3 days in a month<br>6. Once a month<br>7. Less than once a month<br>8. Not in the last 12 months, have I stopped drinking<br>9. Never or just a few sips to taste it throughout life	Alcohol consumption	1. Yes: Options 1 to 6<br>2. No: Option 7 to 9
Could you tell me if you smoke?	1. Yes, I smoke daily<br>2. Yes, I smoke, but not daily<br>3. I don't currently smoke but have smoked before<br>4. I neither smoke nor have I ever smoked regularly	Active smoking	1. Yes: Options 1 and 2<br>2. No: Options 3 and 4

1. Could you tell me how tall you are, approximately, without shoes?<br>2. Could you tell me your weight, approximately, without shoes and clothes?	Body mass index is calculated with the formulae:<br>Weight in kg/ (Height in meters)^2	Body mass index	1. <25<br>2, 25-29.9<br>3. ≥30

COPD, Chronic obstructive pulmonary disease.
